# Role of Qualified Exercise Professionals in Medical Clearance for Exercise: Alberta Cancer Exercise Hybrid Effectiveness-Implementation Study

**DOI:** 10.3390/cancers17172873

**Published:** 2025-09-01

**Authors:** Margaret L. McNeely, Tanya Williamson, Shirin M. Shallwani, Leslie Ternes, Christopher Sellar, Anil Abraham Joy, Harold Lau, Jacob Easaw, Adam Brown, Kerry S. Courneya, S. Nicole Culos-Reed

**Affiliations:** 1 Department of Physical Therapy, University of Alberta, 2-50 Corbett Hall, Edmonton, AB T6G 2G4, Canada; sshallwa@ualberta.ca (S.M.S.); lternes@ualberta.ca (L.T.); csellar@ualberta.ca (C.S.);; 2 Department of Oncology, University of Alberta, 11560 University Avenue NW, Edmonton, AB T6G 1Z2, Canada; anil.joy@ahs.ca; 3 Faculty of Kinesiology, University of Calgary, 2500 University Dr. NW, Calgary, AB T2N 1N4, Canada; willt@ucalgary.ca (T.W.); hlau@ucalgary.ca (H.L.); nculosre@ucalgary.ca (S.N.C.-R.); 4 Medical Oncology, Cross Cancer Institute, 11560 University Ave, Edmonton, AB T6G IZ2, Canada; jay.easaw@ahs.ca; 5 Arthur J. E. Child Comprehensive Cancer Centre, 3395 Hospital Drive NW, Calgary, AB T2N 5G2, Canada; 6 Department of Oncology, University of Calgary, 2500 University Dr. NW, Calgary, AB T2N 1N4, Canada; 7 Faculty of Kinesiology, Sport, and Recreation, University of Alberta, 1-113 University Hall, Edmonton, AB T6G 2H9, Canada; kerry.courneya@ualberta.ca

**Keywords:** cancer survivorship, exercise, physical activity, screening, triage, implementation, knowledge translation

## Abstract

Background: Current guidelines endorse the integration of exercise into cancer care. The diagnosis of cancer and its treatment, however, may introduce factors that make exercise engagement difficult, especially for individuals with advanced stages of disease. In this paper, we describe the baseline screening and triage process implemented for the Alberta Cancer Exercise (ACE) hybrid effectiveness-implementation study and share findings that highlight the multifaceted complexity of the process and the direct role of the clinical exercise physiologist (CEP). Methods: ACE was a hybrid effectiveness-implementation study examining the benefit of 12-week cancer-specific community-based exercise program. The ACE screening process was developed by integrating evidence-based guidelines with oncology rehabilitation expertise to ensure safe and standardized participation across cancer populations. The screening process involved four steps: (1) a pre-screen for high-risk cancers, (2) completion of a cancer-specific intake form and the Physical Activity Readiness Questionnaire for Everyone (PAR-Q+), (3) a CEP-led interview to further evaluate cancer status, cancer-related symptoms and other health issues (performed in-person or by phone), and (4) a baseline fitness assessment that included measurement of vital signs. Results: A total of 2596 individuals registered and underwent prescreening for ACE with 2570 (86.6%) consenting to participate. After full screening including the baseline fitness testing, 209 participants (8.1%) were identified as requiring further medical clearance. Of these, 191 (91.4%) had either a high-risk cancer, metastatic disease or were in the palliative end-stage of cancer, and 161 (84.3%) reported cancer-related symptoms potentially affecting their ability to exercise. In total, 806 (31.4%) participants were triaged to CEP-supervised in-person programming, 1754 (68.2%) participants to ACE community programming, and 8 (0.3%) specifically to virtual programming (post-COVID-19 option). Conclusions: The findings highlight the complexity and challenges of the screening and triage process, and the value of a highly trained CEP-led iterative approach that included the application of clinical reasoning.

## Simple Summary

Current guidelines support integrating exercise into cancer care; however, the cancer diagnosis and treatment-related symptoms can make engagement difficult. The Alberta Cancer Exercise (ACE) hybrid effectiveness-implementation study examined a 12-week cancer-specific, community-based exercise supervised program and implemented a four-step screening process: (1) pre-screen for high-risk cancers, (2) intake form and physical activity readiness questionnaire, (3) clinical exercise physiologist (CEP)-led interview, and (4) baseline fitness assessment. Of 2596 individuals registered, 2570 (86.6%) consented; 877 (34.1%) were medically referred and 1693 (65.9%) self-referred to ACE. Following full screening, 209 (8.1%) required further medical clearance, with most having high-risk or metastatic disease. Many participants in ACE had multiple health and symptom issues related and unrelated to cancer, with common ones occurring in many different combinations. Findings highlight the multifaceted complexity of screening and triage, and the role of the CEP in medical clearance for exercise.

## 1. Introduction

An estimated 2 million North Americans were diagnosed with cancer in 2024, with a significant rise in cancer incidence and mortality due to population aging [1]. Improvements in early detection and more effective locoregional and systemic therapies [2] have resulted in more people living with and beyond cancer [3]. Despite promising cancer outcomes, many individuals struggle to recover from the persistent effects of cancer and its treatment, while others are living in a state of chronic incurable cancer that is controlled or managed with ongoing or periodic treatment [3,4,5,6]. Thus, there is a growing need for interventions to address the physical functioning and health-related quality of life of individuals living with, and beyond cancer [7,8,9,10].

Exercise can help individuals with and beyond cancer manage late and long-term effects, and regain and optimize physical, psychosocial, and vocational functioning following treatment for the disease [7,8]. Specifically, exercise reduces the severity of treatment-related side effects such as pain and fatigue, and benefits psychosocial well-being, including mental and emotional health, and overall quality of life [9]. Moreover, findings of cohort studies indicate that physical activity after a cancer diagnosis is associated with improved survival for breast, colorectal and prostate cancers [11], and a recent multinational randomized trial provided evidence of benefit from structured exercise for both disease-free and overall survival in colon cancer [12]. Collectively, these findings support the role of exercise as both a disease treatment and supportive care intervention [13].

Current guidelines endorse the integration of exercise into cancer care [14]. The diagnosis of cancer and its treatment, however, may introduce factors that make exercise engagement difficult, especially for individuals with advanced stages of disease [14,15]. Moreover, oncology professionals report a lack of knowledge on how to best screen for suitability of exercise [16], with both health professionals and individuals with cancer reporting concerns including fear of injury, worsening symptoms, and skeletal complications [17,18,19]. Screening processes for exercise are commonly used to guide risk stratification and referral to the optimal exercise service or provider based on the individual’s level of need and can prioritize specialist services for those who need it most [20,21]. Clinical exercise physiologists (CEPs), defined as individuals with a relevant exercise-related undergraduate degree and certification in clinical exercise physiology, play a crucial role in determining who can safely proceed to exercise programming, who needs medical clearance before participating, and who may be best served by more closely supervised programming [22]. Medical clearance may be unrestricted, allowing individuals to participate in any type and intensity of exercise, or may be restricted due to significant disease burden (i.e., requiring activity limitations or medically supervised programming) [23]. For some individuals, medical conditions may result in the recommendation to avoid any formal exercise until the condition has abated or is better controlled (i.e., the individual is not cleared for exercise) [23]. Thus, a screening process that includes triage and referral can contribute to a patient-centered, efficient model of care that supports individuals with cancer to engage in exercise [21]. In this paper, we describe the baseline screening and triage process implemented for the Alberta Cancer Exercise (ACE) hybrid effectiveness-implementation study and share findings that highlight the multifaceted complexity of the process and the direct role of the CEPs [24].

## 2. Methods

### 2.1. Study Design

ACE was a hybrid effectiveness-implementation study that opened in January 2017 and completed recruitment in February 2023. The study design and methods have been previously described [25,26]. Here, we outline the specific methods pertaining to the screening process used for exercise clearance and the triage of participants to the most appropriate exercise setting. Ethical approval was received from the Health Research Ethics Board of Alberta: Cancer Committee and all participants were required to provide written informed consent.

### 2.2. Co-Design

ACE was co-designed by exercise oncology researchers, medical and allied healthcare professionals, and individuals with lived experience of cancer. Co-design features proposed by individuals with lived experience of cancer included (1) eligibility of adults with all types and stages of cancer, (2) the ability of individuals with cancer to self-refer to the program, and (3) exercise programming starting at low intensity to build confidence, motivation, and accommodate varying levels of ability [27,28]. In regard to safety, Albertans with lived experience of cancer expressed concerns over risk of injury and the potential for exposure to bacterial/viral infection in public fitness facilities, while Cancer Care Alberta oncology professionals identified the need for baseline evaluation of symptoms/side effects, and physical functioning—factors seen as potentially limiting exercise tolerance [28,29]. Design features that aimed to enhance exercise engagement and safety included: (1) healthcare provider (HCP) approval and guidance for individuals with high-risk cancers or metastatic disease; (2) pre-participation screening and fitness assessment to inform triage to the appropriate level of exercise supervision; (3) ACE classes supervised by exercise professionals with cancer-specific training; (4) provisions for reducing the exercise workload in ACE classes to address “down days”; (5) support for communication, as needed, between the participant’s oncology team and ACE program instructors.

### 2.3. Eligibility Criteria

As per the co-design, eligibility was open to adults with any type and stage of cancer. Individuals could be pretreatment, or receiving cancer treatment, or within three years of completing cancer treatment, or deemed by their referring HCP as needing exercise support due to a long-term or late effect of cancer treatment (e.g., lymphedema, radiation fibrosis syndrome, presence of an ostomy or stoma). Individuals were required to be able to participate in low-intensity exercise at a minimum (i.e., able to do seated exercise yet also transfer independently from sitting to standing and ambulatory with/without a walking aid) and provide informed consent in English (as classes were instructed in English).

### 2.4. Settings

ACE was led by two hub sites overseeing programming in the province of Alberta, one in northern Alberta in Edmonton at the University of Alberta, and one in southern Alberta in Calgary at the University of Calgary. ACE sessions were offered three times a year: winter, spring and fall. Prior to COVID-19, ACE was offered at 18 community locations across seven cities in Alberta including six YMCAs, six municipal fitness centers, three Wellspring Alberta locations (a non-profit cancer support organization), and three academic fitness facilities (University of Alberta, University of Calgary and Lethbridge College) [26]. With the onset of COVID-19 in 2020, virtual exercise assessment and programming were introduced. In-person programming was gradually reinstated (between fall 2021 to winter 2023) to 13 in-person sites, with live stream and virtual group exercise class options (four to six classes per session) continuing to support increased program reach.

### 2.5. Participant Screening for Exercise Safety

The ACE staged screening and triage process was developed by integrating evidence-based guidelines with the research team’s clinical and research expertise. Specifically, the framework was informed by the Canadian Society for Exercise Physiology consensus on physical activity clearance in cancer populations [24,30], as well as key resources from the American College of Sports Medicine [31] and Canadian Society for Exercise Physiology [32]. These resources provided structured risk assessment and clearance criteria, which were adapted to the cancer context to balance participant safety with accessibility. Clinical expertise from experienced oncology rehabilitation and exercise specialists guided refinement of the screening protocol, ensuring feasibility across heterogeneous cancer populations and treatment trajectories. This staged approach enabled standardized yet individualized clearance, to support safe and appropriate exercise clearance.

CEPs with a graduate level degree or certification in clinical exercise physiology [33] and at least five years of clinical experience in exercise oncology were responsible for oversight and coordination of ACE programming, with one CEP leading each hub site. Potentially eligible individuals were screened by the hub CEP for safety and appropriateness of participation in the community-based ACE program. The screening process also served to inform the appropriate level of exercise supervision for each participant with consideration given to the potential for fluctuations in symptoms and performance status for those on active cancer treatment, and/or with advanced and palliative stages of cancer [34].

The screening process involved four steps: (1) a pre-screen for high-risk cancers (introduced in 2018), (2) completion of a cancer-specific intake form and the Physical Activity Readiness Questionnaire for Everyone (PAR-Q+) [32], (3) a CEP-led interview to further evaluate cancer status, cancer-related symptoms and other health issues (performed in-person or by phone), and (4) a baseline fitness assessment that included measurement of vital signs. Further details on the screening considerations for each stage are provided in Table 1.

### 2.6. Data Collection Related to Screening and Triage

At the first stage, potentially eligible participants provided initial consent for the collection of their data related to: (1) their cancer diagnosis, and (2) answering a question on whether they had been told their cancer had spread to another major organ (i.e., bone, brain, liver or lung). Those reporting a high-risk cancer (i.e., sarcoma, lung, head and neck, neurological or pancreatic cancer) or metastatic disease were required to have a referral or obtain approval from their oncologist prior to enrolling in ACE.

Following the pre-screening stage, participants completed the cancer intake form and PAR-Q+ questionnaire [32]. At or prior to the baseline visit, the HUB CEP interviewed the participant to verify questionnaire responses and to probe further into any reported symptoms, side effects, or comorbidities (e.g., in terms of stability and impact on exercise safety/ability). Information gleaned from the interview was also used to determine each participant’s suitability for the planned fitness assessment and to guide the selection of fitness assessment components.

### 2.7. Fitness Assessments (In-Person, Virtual, and Optional)

The in-person fitness assessment included: resting blood pressure, heart rate and oxygen saturation; height and weight (calculation of body mass index), 30 s timed sit-to-stand [35], active shoulder flexion range of motion [36] (flexibility), single-leg balance [37], handgrip strength [38,39,40], and the six-minute walk test [41].

Virtual fitness assessments included: heart/pulse rate (self-measured), two-minute step test [42]; 30 s sit-to-stand [35], shoulder flexion range of motion [36] (flexibility), and single-leg balance [37]. Where possible, data were collected on blood pressure and body weight (if home devices were available).

Where equipment, time and resources allowed, optional tests were offered to participants including: (i) one-repetition maximum bench press (in-person) for upper body muscular strength; (ii) one-repetition maximum leg press (in-person) for lower body muscular strength; (iii) sit-and-reach test (in-person) or single-leg sit-and-reach test (virtual) for flexibility; and (iv) plank test (in-person and virtual) for core muscular endurance.

The CEP consulted with the participant’s oncologist or family physician on any screening or fitness assessment finding potentially requiring further medical evaluation (e.g., bone pain, high resting blood pressure, cognitive issues) and/or referral to oncology rehabilitation services (e.g., poor mobility). The CEP interpreted findings from both the screening and fitness assessment to triage the participant to the appropriate ACE class.

### 2.8. Planned Exercise Intervention

A primary feature of ACE, and a consideration in the exercise clearance and triage process, was that the ACE prescription started with low-intensity exercise and slowly progressed to moderate intensity over the 12-week program duration. Participants attended twice weekly for 60 min per session (starting at approximately 3–4 metabolic equivalent units per session, or approximately 180–240 MET-mins per week) for a 12-week period. Low-intensity options were provided at each session to address fluctuations in energy levels and symptoms [25]. The multimodal prescription comprised aerobic, resistance, balance, and flexibility exercises delivered either as circuit-type classes in an in-person studio setting/virtually, or as group-based personal training conducted in the site’s fitness center. The exercise sessions were conducted in small groups of 8 to 15 participants under the direct supervision of a community-based ACE-trained exercise specialist. Active support and ongoing mentoring by the CEP were provided to the community-based exercise specialists at the participating community location for the duration of the 12-week program. In 2018, we introduced CEP-supervised programming with smaller group sizes (e.g., 3–7 participants) to provide a higher level of exercise tailoring and monitoring for participants with complex presentations related to high symptom burden, advanced complex cancer, or metastatic disease.

## 3. Results

**Figure 1:**
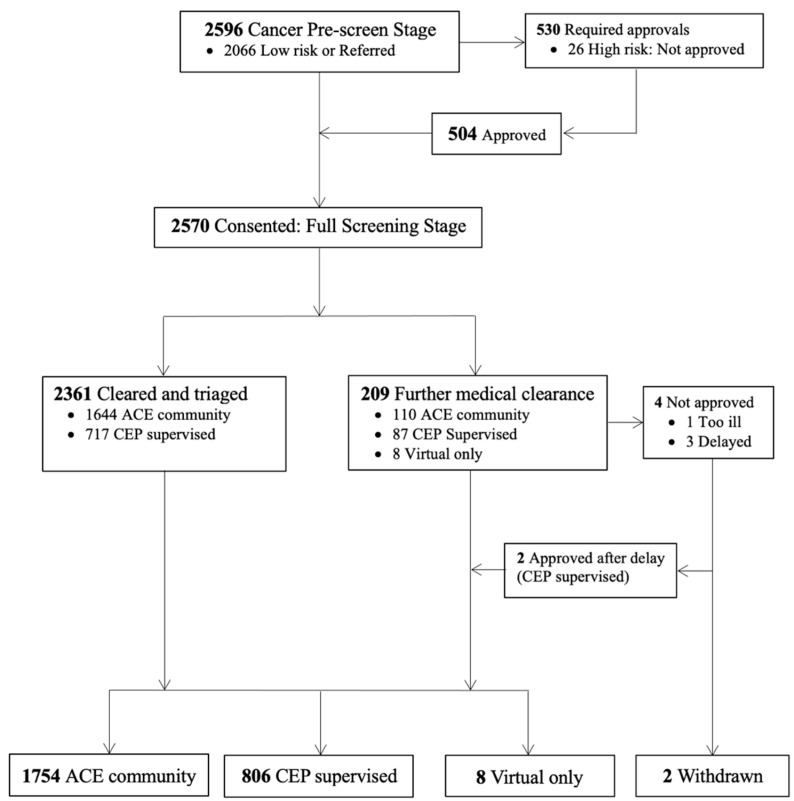
Flow of Participants through the Screening and Triage Process.

The ACE study flow chart for the baseline characteristics has been reported elsewhere [26]. Briefly, a total of 2966 individuals with cancer were referred or self-referred to the ACE program. Of these, 370 individuals were delayed or opted not to proceed after the initial registration phase. We conducted pre-screening on 2596 individuals (87.5%) who were referred or had self-referred to the program, with 2570 (86.6%) consenting to participate in the ACE program (Figure 1). Five hundred and thirty individuals (20.4%) who had self-referred were identified as having a high-risk/metastatic cancer requiring HCP approval prior to enrolment in ACE. Of these participants, 26 (5%) were not approved to participate in ACE due to advanced unstable disease. After full screening including the baseline fitness testing, 209 participants (8.1%) were identified as requiring further medical clearance, with 205 (98%) subsequently cleared to participate. Four (2%) participants were not approved to take part in ACE at the time; however, two (1.1%) were later approved following the initial delay (i.e., for a subsequent ACE session). In total, 806 (31.4%) participants were triaged to CEP-supervised in-person programming, 1754 (68.2%) participants to ACE community programming, and 8 (0.3%) specifically to virtual programming (post-COVID-19 option).

Of participants enrolling, 877 (34.1%) were referred by their HCP team (i.e., oncologist, nurse practitioner or cancer physiatrist) and 1693 (65.9%) self-referred to the program. Although a larger proportion of participants self-referred to the program (Table 2), participants in Calgary were significantly more likely to be directly HCP-referred than participants from Edmonton or northern Alberta (*p* = 0.002). Across all locations, female participants (*p* = 0.01), and participants reporting completion of a graduate degree (*p* < 0.001) were also more likely to be referred.

On the cancer screening intake, individuals diagnosed with breast cancer were significantly more likely to be referred by their HCP team, whereas those with digestive and neurological cancers were more likely to self-refer to the program (*p* < 0.004) (Table 3). Forty-nine percent of participants were undergoing active cancer treatment at the time of enrolment, with chemotherapy (18.6%), hormone therapy (22.2%) and targeted/biological therapy (8.9%) as the most common treatments. Fifty-seven percent of participants self-reported one or more cancer-specific side effects that could potentially interfere with their ability to exercise, with the top three including fatigue (38.3%), muscle and joint issues (31.1%), and peripheral neuropathy (23.0%).

On the PAR-Q+ questionnaire, 1470 (57.2%) participants specified one or more non-cancer health issue, with 497 (19.1%) reporting difficulty controlling the condition or disease (Table 4). Significantly more individuals who self-referred reported one uncontrolled health issue; however, there were no significant differences between referral type for those with more than one uncontrolled health issue. Arthritis was the most reported non-cancer health issue (1168; 45.5%), followed by heart disease (659; 25.6%). For those with a heart-related issue, only 61 individuals (2.4%) reported difficulty controlling their condition with medication or other prescribed therapies. A minority of participants self-reported meeting recommended levels of physical activity, with 796 (31%) categorized as insufficiently active and 1195 (46.6%) as sedentary.

At the fitness testing stage (Table 5), 205 (8.0%) had a body mass index associated with potential health risks and low exercise tolerance [43,44,45]. Seven hundred and twelve (27.7%) had one or more vital signs outside of the recommended range for exercise testing or training [23], with a significantly higher percentage seen in those who had self-referred to the program. An elevated resting heart rate was the most common abnormal vital sign (571; 22.2%) followed by low systolic blood pressure (453; 19.0%). No serious or minor adverse events occurred associated with the baseline fitness testing stage.

**Figure 2:**
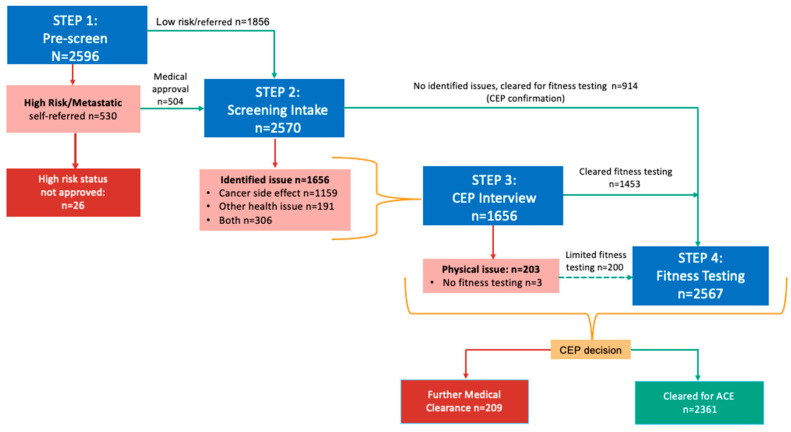
Process and Participant Flow across Screening Stages.

Figure 2 shows the numbers evaluated at each stage of the screening, highlighting the complexity of the exercise clearance process. A total of 2361 (91.9%) participants were cleared for participation in ACE, while 209 (8.1%) required further medical clearance or guidance.

In most cases, the decision to obtain further medical clearance was due to multiple factors potentially influencing exercise tolerance (Table 6). Of note, of the 209 participants identified as requiring further medical clearance, 191 (91.4%) had either a high-risk cancer, metastatic disease or were in the palliative end-stage of cancer (all either referred or having oncologist approval after self-referral), and 161 (84.3%) reported cancer-related symptoms potentially affecting their ability to exercise. For participants with early-stage disease, a high percentage (94.4%) were insufficiently active or sedentary, with one or more vital sign outside of the recommended level. Participants in Edmonton were more likely to require further medical clearance when compared to all other sites (*p* < 0.001).

## 4. Discussion

The benefits of exercise outweigh the risks of participation for most people in the general population [46]. Although less is known about safety of exercise in cancer, especially when the disease is advanced, the overriding message is that exercise is similarly safe and beneficial [14,15]. While physician clearance prior to exercise is standard practice, oncology professionals often lack the knowledge to confidently determine when, how, and which patients to refer to exercise programs [19]. Moreover, requiring a medical evaluation poses a barrier to exercise participation for patients and may not be feasible for medical teams to perform given demands of the busy clinical setting [19]. Instead, we chose to implement a screening process led by CEPs with unique skills, abilities and qualifications to evaluate, prescribe, and monitor exercise and implement lifestyle behavior techniques [47]. Further, the ACE CEPs had training and experience in cancer and the knowledge to tailor exercise to the presenting complexities of the individual participant. These skills were seen as crucial to the efficiency of the screening and triage process, and for ensuring appropriate referral [22]. By integrating the CEP into the decision-making process, we were able to leverage the exercise-specific expertise of the CEP and the knowledge of the oncology team relative to the participant’s diagnosis and symptoms [22,48]. This approach allowed us to decrease the burden on the medical team and remove barriers to enrolment into ACE [48].

A primary finding of the ACE screening and triage process was the need for an iterative approach to unravel the complexity of decision-making around exercise suitability—suggesting that in the cancer setting this process is both an art and science [23]. The four-step approach helped to identify participants requiring further medical clearance or guidance and facilitated focused communication with oncology care teams. We used structured intake forms followed by a CEP-led call/interview with the participant that served to open discussion on presenting issues and ease participant concerns over exercise [49,50]. For the triage process (Figure 2), the CEP made a decision based on the participant’s overall presentation and context (i.e., participant preference for location, availability of resources) and consulted as needed with the oncology team and/or primary physician. The triage also involved decisions on the optimal level of exercise supervision, group size, and the type of training (e.g., circuit vs. where available, group personal training). Moreover, modifications to the prescription could be made according to individual participant’s needs.

With our co-design approach, we adopted an inclusionary enrolment focus, while also aiming to take reasonable steps to ensure the safety of participants and glean information to optimize the exercise experience. Several cancer-specific algorithms have been published to identify exercise-related risks and provide triage recommendations—tools that were not in existence when we first started ACE [20,51,52,53]. Whereas the first step in our ACE screening process involved examining cancer-related factors and the potential for cancer treatment toxicity and symptom burden, published algorithms commonly start by first assessing the individual’s physical activity level and comorbid disease status. These algorithms consistently recommend medical clearance for those who are inactive, have existing comorbid disease, and plan to take part in exercise of moderate or higher intensity. Consideration of these non-cancer-related factors alone would have required medical clearance for the majority (1458; 56.7%) of ACE participants, a barrier to participation we were able to avoid with our supervised format and low-intensity starting point of exercise. In hindsight, while these screening algorithms would have been helpful to us in establishing our ACE process, we also note some limitations in their use. First, we found that there was considerable heterogeneity among participants in cancer stage, type, treatment effects, timing related to treatment, and comorbidities, which added complexity to decision-making. Second, many factors such as the potential for evolving or fluctuating symptoms, and/or unpredictable declines in exercise tolerance due to cancer treatments, side effects, or disease progression, are not easily reduced into an algorithm [23]. Third, triage recommendations include referral to services such as cancer rehabilitation for supervised exercise, services not consistently available nor accessible to everyone across socioeconomic levels and geographical locations. While there may be options in the future for artificial intelligence derived from models to aid in the process, some flexibility in decision-making is needed given the individual’s context and presenting health status, extending beyond that of a static algorithm.

Surveys reveal many individuals with cancer have positive perceptions of exercise and are motivated to adopt healthy behaviors after a diagnosis [22]. Thus, the diagnosis of cancer is commonly referred to as a ‘teachable moment’ to introduce health behavior change [54]. While in theory, those who self-refer to exercise are possibly more physically able and motivated [55], we found surprisingly few differences in cancer type, treatments, and symptoms between individuals who were referred and those who self-referred to the program. Participants who self-referred to ACE, however, were significantly more likely to report an uncontrolled non-cancer health issue and to present with a vital sign outside of the recommended level for exercise, suggesting poorer overall health status. Moreover, while a larger proportion of individuals self-referred to ACE, participants who were female, with a diagnosis of breast cancer, graduate level education, and living in Calgary were more likely to be referred by their oncology team. These findings are likely attributable to the tumor-specific exercise oncology research conducted across the province [56,57,58,59], clinical relationships with research teams at the two hub sites, and the availability of cancer physiatry services located in Calgary.

### 4.1. Limitations

A primary limitation of the ACE hybrid effectiveness-implementation study is the lack of a control group to allow for the future investigation of our screening and triage findings relative to usual care. Moreover, 99.0% of ACE participants were at a contemplation or higher stage of behavior change [26], representing a sample likely more motivated to engage in exercise. As many individuals diagnosed with cancer report being sedentary [60,61], strategies such as motivational interviewing are suggested to target the large proportion disinclined to engage in exercise [62]. A further limitation of ACE is the use of CEPs with access to clinical information and experience working in cancer, factors that limit generalizability to other settings, but also highlight an ideal program characteristic supporting delivery of high-quality screening for individuals with cancer. Lastly, research is needed to validate our screening process by assessing its ability to identify those in need of medical clearance and to determine which participants are most likely to complete and benefit from the program.

### 4.2. Patient Partner Perspective

Our patient research partners contributed meaningfully to the design and delivery of the ACE program, advocating for inclusion of individuals with all types and stages of cancer, and the self-referral option. They report finding the screening and triage process to be responsive, compassionate, and attuned to the real-world needs of individuals with cancer, which helped bridge the gap between clinical research and patient realities. Participating in ACE was seen to support their recovery in the cancer journey while also arming them to be advocates for the inclusion of exercise as a vital component of care for others facing similar diagnoses.

## 5. Conclusions

In summary, many participants in ACE had multiple health and symptom issues related and unrelated to cancer, with common ones occurring in many different combinations. The findings highlight the complexity and challenges of the screening and triage process, and the value of a highly trained CEP-led iterative approach that included the application of clinical reasoning. The ACE CEPs took on greater responsibility at both hub sites for the screening and triage process, working in conjunction with information and guidance from the oncology team as needed. This transdisciplinary team approach supported participants to access exercise oncology resources.

## Figures and Tables

**Table 1:** Screening Steps and Considerations.

Steps in Screening	Screening Considerations	Action Taken if Risk Identified	Comments
Step 1: Pre-screen for high-risk cancers	Identify individuals reporting a high-risk cancer type (i.e., head and neck cancer, neurological, lung, pancreatic or multiple myeloma) or reporting local or distant metastatic disease	HCP * guidance on appropriateness for program and recommended level of exercise supervision	This step was instituted after the first year of ACE programming to avoid delays in baseline fitness assessment due to concerns over high-risk status
Step 2: Screening intake	CEP review of cancer intake and PAR-Q+ questionnaires to identify cancer-related and/or current conditions respectively	Further HCP * guidance/clearance as needed before conducting baseline fitness assessment	Further questions were added to expand the intake for cancer category types and sub-types; and for those reporting >1 cancer diagnosis
Step 3: CEP-led interview with participant	In-person/virtual or phone interview with CEP to review screening findings with the participant to determine status, and the need for further medical clearance	HCP * guidance if further cancer-related issues identified on interview Primary care provider or specialist guidance on non-cancer related issues	Informed level of supervision, need for tailored or personalized exercise prescription (e.g., monitoring of shortness of breath; modification to exercise due to arthritis in knee)
Step 4: Physical fitness assessment	Evaluate physical function, mobility and fitness. Ensure there are no underlying conditions (e.g., hypertension, tachycardia, poor mobility) that may be made worse by engaging in exercise	HCP * or primary care provider (if discharged from cancer care) contacted if identified issues requiring further medical guidance or investigation	Identify need for medical follow-up or monitoring of vitals; inform tailoring of exercise based on results

* HCP: oncologist, physiatrist, nurse practitioner or point-of-care medical provider.

**Table 2:** Demographic characteristics of the Alberta Cancer Exercise cohort at Baseline: Overall (N = 2570) and by referral method.

Demographic Characteristic	Total Cohort (N = 2570)		Self-Referral (n = 1693)		Medical Referral (n = 877)	
	Mean/n	SD/%	Mean/n	SD/%	Mean/n	SD/%
Age in years, mean, SD	57.8	12.0	57.9	12.1	57.7	11.8
17 to 39 years (AYA)	190	7.4%	127	7.5%	63	7.2%
40 to 64 years	1582	61.6%	1030	60.8%	552	62.9%
65+ years	798	31.1%	536	31.7%	262	29.9%
Biological Sex						
Female	1832	71.3%	1179	69.6%	653	74.5% ^†^
Male	738	28.7%	514	30.4%	224	25.5%
Population Group						
European descent	1859	72.3%	1212	71.6%	647	73.8%
Other	711	27.7%	481	28.4%	230	26.2%
Urban Site						
Calgary	1149	44.7%	714	42.2%	435	49.6% ^†^
Edmonton	1122	43.7%	764	45.1%	358	40.8% ^†^
South urban sites	122	4.7%	85	5.0%	37	4.2%
North urban sites	177	6.9%	130	7.7%	47	5.4% ^†^
Marital status						
Never married	261	10.2%	162	9.6%	99	11.3%
Married/common-law	1858	72.3%	1233	72.9%	624	71.2%
Divorced/separated	339	13.2%	214	12.6%	125	14.3%
Widowed	112	4.4%	83	4.9%	29	3.3%
Highest level of education						
High School * or less	823	32.0%	579	34.2%	244	27.8%
University/college	1334	51.9%	868	51.3%	466	53.1%
Graduate school	412	16.0%	245	14.5%	167	19.0% ^†^
Missing/not reported	1	0%	1	0%	-	-
Income						
<$60,000	767	43.6%	518	30.6%	249	28.4%
$60,000 to $99,999	716	27.9%	458	27.1%	258	29.4%
≥$100,000	810	31.5%	533	31.5%	277	31.6%
Missing/not reported	277	10.8%	184	10.9%	93	10.6%

^†^ significant difference between referral groups; * Highschool +/− some university or college.

**Table 3:** Cancer Intake at Baseline: Overall (N = 2570) and by Referral Type.

Cancer and Treatment Characteristics	Total Cohort (N = 2570)		Self-Referral (n = 1693)		Medical Referral (n = 877)	
	Mean/n	SD/%	Mean/n	SD/%	Mean/n	SD/%
PRE-SCREEN (self-report)						
High-risk cancer type *	455	17.7%	312	18.4%	143	16.3%
Metastatic disease	450	17.5%	306	18.1%	144	16.4%
High-risk and metastatic	91	3.5%	57	3.4%	34	3.9%
CANCER INTAKE FORM						
Single primary cancer	2392	93.1%	1567	92.6%	823	93.8%
Two/more primary cancers	178	7.0%	126	7.4%	54	6.2%
Primary Cancer Type						
Breast	1167	45.4%	738	43.6%	429	48.9% ^†^
Hematologic	355	13.8%	221	13.1%	134	15.3%
Genitourinary	241	9.4%	163	9.6%	78	8.9%
Digestive	231	9.0%	169	10.0% ^†^	62	7.1%
Head and Neck	159	6.2%	110	6.5%	49	5.6%
Gynecologic	152	5.9%	97	5.7%	55	6.3%
Neurological	93	3.6%	74	4.4% ^†^	19	2.2%
Lung	90	3.5%	62	3.7%	28	3.2%
Other	82	3.2%	59	3.5%	23	2.6%
Cancer Treatment Status						
On treatment	1254	48.8%	823	48.6%	431	49.1%
Off treatment	1316	51.2%	870	51.4%	446	50.9%
Current cancer treatment—type						
Chemotherapy Treatment	477	18.6%	324	19.1%	153	17.4%
Radiation Therapy	134	5.2%	84	5.0%	50	5.7%
Hormonal Therapy	570	22.2%	364	21.5%	206	23.5%
Targeted/Biological Therapy	229	8.9%	152	9.0%	77	8.8%
Other treatment	42	1.6%	26	1.5%	16	1.8%
Treatment-related effects potentially interfering with exercise						
Number reporting ≥1 effects	1465	57.0%	988	58.4%	477	54.4%
Fatigue	984	38.3%	662	39.1%	322	36.7%
Muscle or joint issues	799	31.1%	539	31.8%	260	29.6%
Peripheral Neuropathy	590	23.0%	391	23.1%	199	22.7%
Pain	503	19.6%	337	19.9%	166	18.9%
Weight-related issues	416	16.2%	278	16.4%	138	15.7%
Cognitive challenges	413	16.1%	287	17.0%	126	14.4%
Lymphedema	249	9.7%	185	10.9%	64	7.3%
Bladder/bowel issues	219	8.5%	154	9.1%	65	7.4%
Shortness of breath	189	7.4%	126	7.4%	63	7.2%
Osteoporosis	88	3.4%	62	3.7%	26	3.0%
Communication issues	69	2.7%	42	2.5%	27	3.1%
Cardiac issues	55	2.1%	35	2.1%	20	2.3%
Ostomy issues	33	1.3%	19	1.1%	14	1.6%
Other	182	7.1%	122	7.2%	60	6.8%

* High risk: multiple myeloma, head and neck, lung, pancreatic, and primary brain tumors or confirmed metastatic disease to a distant site or organ; ^†^ significant difference between referral groups.

**Table 4:** PAR-Q+ and Physical Activity at Baseline: Overall (N = 2570) and by Referral Type.

Lifestyle Characteristics	Total Cohort (N = 2570) Mean/n (SD/%)		Self-Referral (n = 1693) Mean/n (SD/%)		Medical Referral (n = 877) Mean/n (SD/%)	
Number with Other Identified Health Issues on PAR-Q+						
One health issue PAR-Q+	886	34.5%	603	35.6%	283	32.3%
≥Two health issues PAR-Q+	584	22.7%	615	36.3%	334	38.1%
One uncontrolled health issue	365	14.2%	265	15.7% ^†^	100	11.4%
≥Two uncontrolled health issues	132	5.1%	81	4.8%	51	5.8%
PAR-Q+ Responses						
Arthritis	1168	45.5%	769	45.4%	399	45.5%
Difficulty controlling	127	4.9%	78	4.6%	49	5.6%
Pain/recent fracture	300	11.7%	192	11.3%	108	12.3%
Heart Disease	659	25.6%	452	26.7%	207	23.6%
Difficulty controlling	61	2.4%	42	2.5%	19	2.2%
Mental Health Issue	388	15.1%	247	14.6%	141	16.1%
Difficulty controlling	60	2.3%	41	2.4%	19	2.2%
Respiratory Disease	304	11.8%	202	11.9%	102	11.6%
Difficulty controlling	31	1.2%	23	1.4%	8	0.9%
Low oxygen levels	12	0.5%	8	0.7%	4	0.5%
Metabolic Disease	250	9.7%	167	9.9%	83	9.5%
Difficulty controlling	45	1.8%	32	1.9%	13	1.5%
Diabetic neuropathy	48	1.9%	37	2.2%	11	1.3%
Prior Stroke	65	2.5%	44	2.6%	21	2.4%
Difficulty controlling	4	0.2%	2	0.1%	2	0.2%
Difficulty walking	8	0.3%	6	0.4%	2	0.2%
Prior Spinal Cord Injury	21	0.8%	13	0.8%	8	0.9%
Fainting/loss consciousness	146	5.7%	105	6.2%	41	4.7%
Other Health Condition	405	16.6%	251	14.8%	154	17.6%
Physical Activity category						
Insufficiently Active	796	31.0%	405	31.3%	391	30.8%
Sedentary	1195	46.6%	616	47.7%	579	45.6%

^†^ significant difference between referral groups.

**Table 5:** Fitness Test Screening Stage: Body Mass Index and Vitals.

Health-Related Fitness Measures	Total Cohort (N = 2570)		Self-Referral (n = 1693)		Medical Referral (n = 877)	
	Mean/No.	SD/%	Mean/No.	SD/%	Mean/ No.	SD/%
Underweight: BMI < 18.5	40	1.6%	25	1.5%	15	1.7%
Obese Class III or higher: BMI ≥ 40	165	6.4%	107	6.3%	58	6.6%
≥1 Vital sign outside recommended level	712	27.7%	497	29.4% ^†^	215	24.5%
High resting heart rate	571	22.2%	395	23.3%	176	20.1%
Resting systolic BP > 160 mmHg	30	1.2%	24	1.4%	6	0.7%
Resting diastolic BP > 100 mmHg	33	1.3%	23	1.4%	10	1.1%
Resting systolic BP < 90 mmHg	453	19.0%	315	20.1% ^†^	138	16.7%
Resting diastolic BP < 60 mmHg	40	1.9%	28	2.0%	12	1.7%
Resting oxygen saturation < 90	8	0.3%	5	0.3%	3	0.3%

^†^ significant difference between referral groups; BP: blood pressure.

**Table 6:** Profile of Participants Requiring Further Medical Clearance/Guidance after Screening.

Profile of Participants	Overall N = 209 * No., (%)	Early-Stage Disease n = 18 No., (%)	High-Risk Status n = 93 No., (%)	Metastatic Disease n = 96 No., (%)	Palliative Stage-Withdrawn n = 2 No., (%)
Cancer Type	Breast: 36 Lung: 17 Digestive: 27 Hematologic: 40 Gynecologic: 6 Genitourinary: 25 Neurologic: 48 Other: 10	Breast: 1 Digestive: 4 Hematologic: 6 Gynecologic: 2 Genitourinary: 2 Other: 3	Lung: 10 Pancreatic: 2 Multiple Myeloma: 33 Neurologic: 48	Breast: 34 Lung: 7 Digestive: 20 Hematologic: 1 Gynecologic: 4 Genitourinary: 23 Other: 7	Breast: 1 Digestive: 1
Self-referred	149 (71.3%)	10 (55.8%)	68 (73.1%)	69 (71.2%)	2 (100%)
On treatment	152 (72.7%)	9 (50.0%)	59 (63.4%)	82 (85.4%)	2 (100%)
Symptoms affecting exercise	172 (82.3%)	11 (61.1%)	76 (81.7%)	83 (86.5%)	2 (100%)
Physical activity level: inactive/ sedentary	177 (85.1%)	17 (94.4%)	78 (83.9%)	80 (84.2%)	2 (100%)
≥1 uncontrolled health conditions	43 (20.6%)	4 (22.2%)	13 (14.0%)	26 (27.1%)	0 (0%)
BMI > 40	12 (5.7%)	1 (5.6%)	3 (3.2%)	8 (8.3%)	-
BMI < 18.5	3 (1.4%)	-	1 (1.1%)	2 (2.1%)	2 (100%)
≥1 Vital sign outside of recommended level	103 (49.3%)	11 (61.1%)	36 (38.7%)	55 (57.3%)	1 (50.0%)

* 4 occurring prior to instituting the pre-screen stage (1 high risk; 3 metastatic).

## Data Availability

The data presented in this study are available on request from the corresponding author due to ongoing data collection and analyses.
